# Morphological and genetic characteristics of F_1_ hybrids introgressed from *Brassica napus* to *B. rapa* in Taiwan

**DOI:** 10.1186/s40529-019-0279-5

**Published:** 2020-01-21

**Authors:** Yuan-Kai Tu, Han-Wei Chen, Kuang-Yu Tseng, Yen-Chun Lin, Bo-Jein Kuo

**Affiliations:** 1 Division of Biotechnology, Taiwan Agricultural Research Institute, No.189, Zhongzheng Rd., Wufeng Dist., Taichung City, 41362 Taiwan (R.O.C.); 2 Department of Agronomy and Innovation and Development Center of Sustainable Agriculture (IDCSA), National Chung Hsing University, No.145 Xingda Rd., South Dist., Taichung City, 40227 Taiwan (R.O.C.)

**Keywords:** Introgression, *Brassica napus*, *Brassica rapa*, Morphology, SRAP marker, Genetic similarity

## Abstract

**Background:**

Unintentional introgression from genetically modified (GM) oilseed rape (*Brassica napus*) to a relative is inevitable in the open field. A feasible and practical strategy for restricting the spread of GM offspring is to set a reasonable isolated distance between GM *B. napus* and the relatives. To define the isolated distance, a pollen donor/recipient pair is a prerequisite to conducting the field trial of pollen flow. However, because the cultivation of GM *B. napus* is prohibited in Taiwan, it is difficult to obtain relevant information. Thus, this study explored the morphological and genetic characteristics of five varieties of *B. napus* (donor), three varieties of *B. rapa* (recipient), and the 15 corresponding F_1_ hybrids, aiming to construct phenotypic data and genetic variation data and to select the most appropriate pollen donor/recipient for future field trials of pollen flow.

**Results:**

The genome size of all F_1_ hybrids estimated using flow cytometry showed intermediate DNA content between *B. napus* and *B. rapa* varieties. Most of the F_1_ hybrids had intermediate plant height and blooming period, and the rosette leaves type and colors resembled those of *B. napus* varieties. The results of sequence-related amplified polymorphism (SRAP) showed an average of 9.52 bands per primer combination and 67.87 polymorphic bands among the F_1_ hybrid population. Similarity and cluster analyses revealed higher similarity between F_1_ hybrids and *B. napus* varieties than between F_1_ hybrids and *B. rapa* varieties. Furthermore, we identified a specific 1100-bp band (*LOC106302894*) in F_1_ hybrids and *B. napus* varieties but not in *B. rapa* varieties.

**Conclusions:**

The rosette leaves and the DNA marker LOC106302894 observed in F_1_ hybrids are consistent phenotypic and genetic characteristics that can be used to identify the presence of unintentional hybridization from *B. napus* to *B. rapa* in Taiwan. Due to the prohibition of GM crop cultivation, the hybridization system of non-GM *Brassica* species in this study can be utilized as a mimic scheme to conduct pollen flow trials, thus facilitating the determination of the proper isolated distance.

## Background

*Brassica* species, which belong to the family *Brassicaceae*, are cultivated globally for food, oilseed, and manure. The classification of *Brassica* plants has been described by the theory of the triangle of U, which is based on the polyploidization events in *Brassica* species and the chromosomal architecture of genomes. There are six *Brassica* species and three genome types—A (n = 10), B (n = 8), and C (n = 9). The combination of different genomes generates three allotetraploid species [*B. juncea* (AABB, 2n = 36), *B. carinata* (BBCC, 2n = 34), and *B. napus* (AACC, 2n = 38)] originating from natural hybridizations between any two of the three diploid species [*B. rapa* (AA, 2n = 20), *B. nigra* (BB, 2n = 16), and *B. oleracea* (CC, 2n = 18)] (Nagaharu 1935).

In Taiwan, *B. rapa* species are widely cultivated as food and green manure. *B. napus* a major oilseed crop in the world, along with *Glycine max*, *Arachis hypogaea*, and *Helianthus annuus*. Using genetic engineering techniques such as introducing foreign genes, gene silencing, and gene editing, genetically modified (GM) oilseeds of *Brassica* species provide very efficient methods to generate new traits such as herbicide tolerance, fatty acid composition changes, increase in biotic/abiotic stress resistance, and fertility restoration (Leckband et al. 2002; Kayum et al. 2015). However, gene transfer from GM crops to their wild species (or relatives) in the open field may result in the problem of cross-pollination.

GM and non-GM crops are identical except for the incorporated foreign DNA and the target trait. Nevertheless, the crucial issue for GM crops is gene flow, which may unintentionally confer herbicide resistance in compatible relatives. The frequencies of spontaneous hybridization between *B. napus* and *B. rapa* ranged from 13 to 93% according to various field experiments in Denmark (Jørgensen and Andersen 1994). Regarding the gene flow from the GM *B. napus* to the non-GM *B. rapa*, Halfhill et al. (2002) indicated the hybridization rate ranged from 0.7% to 16.9, in which *Bacillus thuringiensis* (*Bt*)-transgenic *B. napus* and non-GM *B. rapa* were used as male and female parents under field conditions. Other studies on gene flow from GM *B. napus* to non-GM *B. rapa* have been conducted in the United Kingdom and Japan (Warwick et al. 2008; Aono et al. 2011; Luijten et al. 2015). Moreover, the seeds of GM *B. napus* with herbicide-resistant traits were found along the roadside and railway lines in Japan and Canada, and these spilled seeds survived and crossed with *B. rapa* relatives (Yoshimura et al. 2006; Nishizawa et al. 2016). Furthermore, the escapes of GM *B. napus* event GT73 were reported around the port area and railway in Switzerland, where no cultivation and importation of GM *B. napus* were allowed (Hecht et al. 2014). The abovementioned studies have revealed that cross-pollination between GM *B. napus* and wild-type *B. rapa* is possible and may adversely affect not only the ecological diversity but also the agri-product supply chain (Zdjelar et al. 2011). To the best of our knowledge, no commercial GM crops have been cultivated in open fields in Taiwan (Kuo 2012). However, 11 GM *B. napus* events (RF3, MS8, MS11, GT73, MON88302, 73496, MS8xRF3, MS8xRF3xGT73, MON88302xMS8xRF3, MON88302xRF3, and 73496xRF3) are allowed to be imported to Taiwan for industry purposes (Ministry of Health and Welfare 2019). Discrimination between *B. napus* and *B. rapa* is feasible according to specific morphological traits, but environmental conditions could drastically influence crop morphology. It is thus necessary to establish an efficient method for recognizing the hybrids between *B. napus* and *B. rapa* varieties in an early stage to prevent and monitor the occurrence of unintentional introgression in Taiwan.

Several techniques have been utilized for identifying interspecific F_1_ hybrids. For example, the combination of flow cytometry (FCM) and amplified fragment length polymorphism markers was used to examine the relationship of *B. napus* with *B. rapa* and introgressed individuals (Warwick et al. 2003; Luijten et al. 2015). Chromosomal pairing and recombination at meiosis in *B. rapa* (AA) crossed with *B. napus* (AACC) were observed using BAC-based FISH (Fluorescence in situ hybridization) and confirmed by a chromosome-specific marker genome for A or C chromosome, respectively (Leflon et al. 2006). Molecular markers for selecting different *Brassica* species and evaluating the genetic relationships have been developed (Liu and Wang 2006; Ananga et al. 2008; Christensen et al. 2011; Ali Turi et al. 2012; Ahmad et al. 2014). The sequence-related amplified polymorphism (SRAP) marker is a powerful tool because of its simplicity, reproducibility, cost-effectiveness, and multi-loci assessment (Li and Quiros 2001; Ahmad et al. 2014). SRAP markers were developed to amplify open reading frames (ORFs) with forward and reverse primers containing GC- and AT-rich sequences near the 5ʹ and 3ʹ ends, respectively, to identify genome variations in ORFs. By using SRAP marker analysis, F_1_ hybrids have been characterized in various crops, including *Paeonia*, *Arachis*, and *Coffea* genus hybrids (Hao et al. 2008; Ren et al. 2010; Mishra et al. 2011).

Recently, Hong et al. identified interspecific hybrids derived from crossing the *B. napus* variety “Deza oil No. 18” with *B. rapa* variety “Nongxing 80 days” (Hong et al. 2016). Phenotypic traits such as leaf shape and lobed leaf of the hybrids were closer to those of the *B. napus* variety “Deza oil No. 18.” SRAP analysis suggested that at least five polymorphic markers could be used to discriminate F_1_ hybrids in the greenhouse and open-pollination fields (Hong et al. 2016). Thus, the SRAP marker can be a potential method to examine the genetic diversity, as it is stable, reproducible, and simple to analyze.

In this study, we selected 16 SRAP primer sets to analyze the possible genetic relationship among five *B. napus* varieties, three *B. rapa* varieties, and the corresponding fifteenth interspecific F1 hybrids. Using FCM, morphological measurements, and SRAP analysis, we conducted a comprehensive evaluation of morphological and genetic characteristics of (parents and) F_1_ hybrids derived from *B. rapa* (♀) × *B. napus* (♂) in Taiwan. The results could be used to develop a robust and highly reproducible system for the early identification of F_1_ hybrids introgressed from *B. napus* to *B. rapa* in Taiwan.

## Materials and methods

### Plant materials and growth condition

We selected three *B. rapa* varieties (“Nongxing 80 days,” “Wansheng rape,” and “Edible rape”), which are the common vegetable cultivars for food or green manure in Taiwan. The *B. napus* varieties, which are used for producing edible rapeseed oil, included four cultivars imported from China [“Deza oil No. 18” (a genetic male sterility double-cross variety), “Gueiza No. 4” (a double-cross variety), “Zhong oil No. 36” (a cytoplasmic male sterility triple-cross variety), and “Wan oil No. 25” (a genetic male sterility double-cross variety)] and one cultivar “FTHEB 1001,” which is a synthetic variety in Taiwan (Hsieh et al. 2013). To generate a specific combination of F_1_ hybrids, *B. rapa* (pollen recipient) was pistil pollinated by the stamen of *B. napus* (pollen donor). All the seedlings were grown in pots in the greenhouse at 25 °C with a natural photoperiod.

### Morphology investigation

The morphological traits of each variety of the 30 plants were investigated in the field. In brief, the morphological test in this study was based on the International Union for the Protection of New Varieties of Plants (UPOV) Test Guidelines for DUS (distinctness, uniformity, and stability) testing’; the guidelines were specifically used for *B. napus* L*. oleifera* (reference number TG/36/6) and *B. rapa* L. *var. silvestris* (Lam.) Briggs (reference number TG/185/3). The morphology of the rosette leaves was observed during the entire growth period in the field, along with an additional examination in the greenhouse. The morphology of stem leaves and flowers was examined from the period of bud formation to blooming. Rosette and stem leaves were grouped based on color using the Royal Horticultural Society (RHS) Colour Chart: 1 (shine green/143A), 2 (dull green/138B), and 3 (dark green/137A or N137A). Lobed leaf and wax texture were indicated as 1 (absent) or 2 (present). Plant height was measured at complete flowering, when the lower siliques were elongating. The blooming period was recoded from the first flowering to wilting of all flowers.

### FCM procedures

The Ploidy Analyser (PA) flow cytometer (Sysmex Partec GmbH, Germany) was used for genome size analysis, and *B. rapa* “Nongxing 80 days” was used as a reference in this study. For nuclei extraction, young seedlings with the first expanded leaves and a height of approximately 15 cm were collected. The rosette leaves (1 cm^2^) were chopped into small pieces using a razor blade with 0.2 mL of CyStain UV Precise T solution A (Sysmex Partec GmbH) in a Petri dish. The suspension was filtered, and 0.8 mL of CyStain UV Precise T solution B (Sysmex Partec GmbH) was then added. After staining with solution B for at least 3 min, the final suspension was injected into the flow of sheath fluid to measure the relative amount of nuclear DNA. Distribution and relative fluorescence intensity of the F1 hybrids, *B. rapa* varieties, and *B. napus* varieties were analyzed in three replicates.

### DNA extraction

The samples of 0.1-g young leaves from different seedlings were harvested and lyophilized in liquid nitrogen and preserved at − 80 °C. DNA was extracted using the GeneMark Plant Genomic DNA Purification Kit (GMbiolab, Taiwan). The concentration and purity of DNA were determined by the OD_260_ and OD_260_/OD_280_ values measured by the NanoDrop ND-2000 Spectrophotometer (Thermo Scientific, Wilmington, USA). The DNA samples were diluted to 100 ng/μL and then stored at − 20 °C.

### SRAP marker analysis

Based on primer designs by Li and Quiros (2001), 16 primer set combinations using four forward and four reverse primers were employed in the SRAP marker analysis (Table 1). The primers were synthesized by Jier Sheng Biotechnology Co. Ltd. (Taichung, Taiwan). PCR amplification was performed with a 25-μL reaction mixture containing 1× Ex Taq buffer (Mg^2+^ plus) (TaKaRa), 0.2 mM dNTPs, 0.6 μM of each forward and reverse primer, 1.5 unit Ex Taq (TaKaRa), and 100 ng of template DNA. The reaction was conducted in a GeneAmp^®^ PCR System 9600 (Applied Biosystems, Foster City, CA, USA). The thermal cycling profile was programmed with an initial denaturation at 94 °C for 5 min; followed by five cycles at 94 °C for 1 min, 35 °C for 1 min, and 72 °C for 1 min; and 35 cycles at 94 °C for 1 min, 50 °C for 1 min, and 72 °C for 2 min. The samples were subjected to final extension at 72 °C for 10 min. The PCR products were separated on 1.8% agarose gel in 1× TAE (Tris–acetate-EDTA) buffer. The gel profile was detected by staining with ethidium bromide, and the bands were visualized under a UV transilluminator (DUT-260; Core Bio System, Seoul, South Korea). The photographs were documented by the Kodak Gel Logic 100 Imaging System (Kodak, Rochester, NY, USA). To test the reproducibility of the SRAP markers, independent PCR amplifications were performed 3–5 times.

**Table 1:** List of primers for SRAP marker analysis and the loc fragment

Forward primers (5′→3′)		Reverse primers (5′→3′)	
me1	TGAGTCCAAACCGGATA	em1	GACTGCGTACGAATTAAT
me2	TGAGTCCAAACCGGAGC	em2	GACTGCGTACGAATTTGC
me5	TGAGTCCAAACCGGAAG	em5	GACTGCGTACGAATTAAC
me6	TGAGTCCAAACCGGACA	em6	GACTGCGTACGAATTGCA
loc-f	TACCACACTCAGACGCAG	loc-r	CTAATCAGGAGTGCGTGC

### Data collection and genetic similarity analysis

The banding pattern of each sample was transformed into a spreadsheet to form a binary matrix. Only distinct, clearly distinguishable, and reproducible fragments were used in the genetic similarity analysis. Each of these fragments was scored independently as 1 (present) or 0 (absent). Polymorphism was calculated on the basis of the proportion of common bands between two accessions, with 0 representing no common bands and 1 representing common bands. The common band between two accessions was identified as the polymorphic band. Each polymorphic band was scored as a single dominant marker (Li and Quiros 2001).

Genetic similarity (GS) among the accessions was calculated using the following equation: GS_i,j_ = 2N_i,j_/(N_i_ + N_j_) (Nei and Li 1979), where N_i,j_ is the number of bands shared by individuals i and j, and N_i_ and N_j_ are the total number of bands in individuals i and j, respectively. Cluster analysis of the matrix of GS was performed through of the unweighted pair group method with arithmetic mean (UPGMA). A dendrogram was constructed using the PAST program, and the Jaccard similarity index was used to calculate GS (Hammer et al. 2001).

### Cloning and sequencing of a specific SRAP marker band

The 1100-bp fragment amplified from the SRAP marker analysis using the primer set me5-em1 was separated through agarose gel electrophoresis. The fragment was cloned into the pGEM-T Easy Vector (Promega) and was sequenced. The sequence was analyzed against the nonredundant database using the basic local alignment search tool program (BLAST).

### Examining the existence of SRAP marker

The primers were designed for the 1100-bp fragment amplified from the SRAP marker analysis using the primer set me5-em1 by the Primer 3.0 software based on the sequencing results. The forward primer loc-f and the reverse primer loc-r are listed in Table 1. *B. tubulin beta*-*6* (*BraTUB6*) was used as the internal control, with BraTUB6f (5ʹ-GTGGAATGGATACCGAAC-3ʹ) as the forward primer and BraTUB6r (5ʹ-GTTGCGTCTTGGTATTGC-3ʹ) as the reverse primer. The thermal cycling profile was programmed to 1 cycle at 94 °C for 5 min, followed by 35 cycles at 94 °C for 30 s, 58 °C for 0.5 s and 72 °C for 50 s, and 72 °C for 7 min as the final extension. The amplified products were examined on the 1.8% agarose gel, as mentioned above.

## Results

### Genome size determination through FCM

**Fig. 1:**
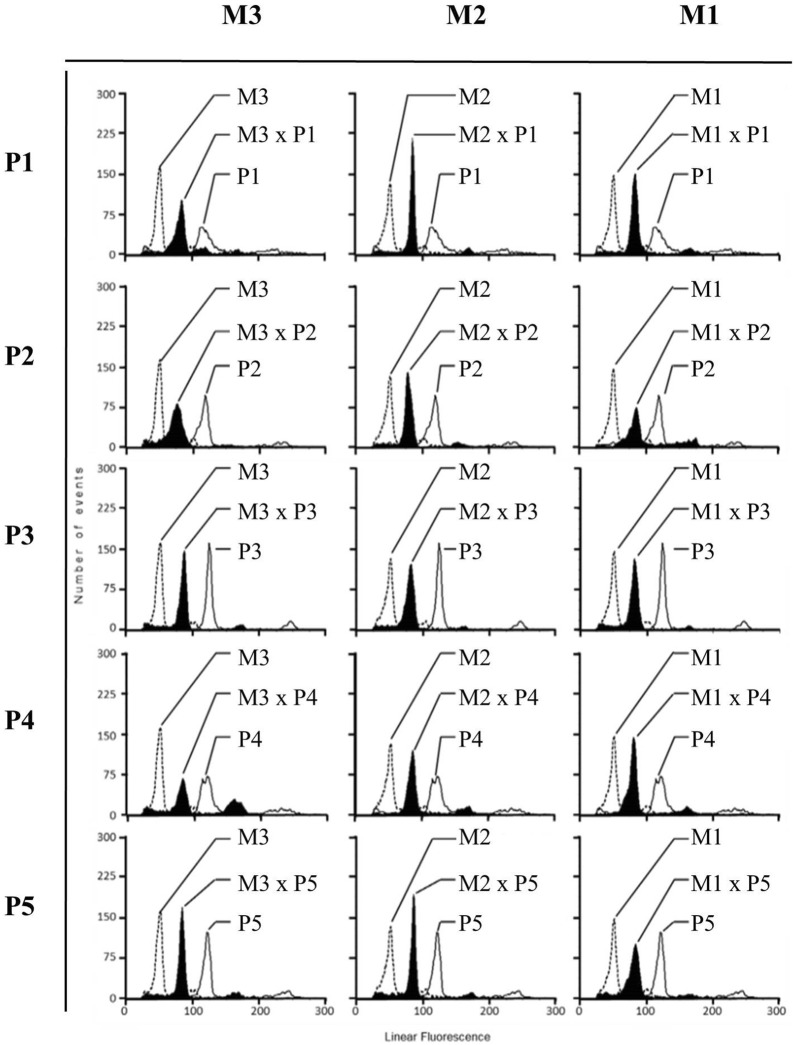
Relative distribution and fluorescence intensity of *B. rapa*, *B. napus*, and F_1_ hybrids. M1: “Nongxing 80 days,” M2: “Wansheng rape,” M3: “Edible rape,” P1: “Deza oil No. 18,” P2: “Gueiza No. 4,” P3: “Zhong oil No. 36,” P4: “Wan oil No. 25,” and P5: “FTHEB 1001.” The internals of F_1_ hybrids are indicated by the black area

Estimation of the genome size between the F_1_ hybrids and their parents was conducted through FCM. The values revealed two distinct sizes with an average of 50 in *B. rapa* and 110 in *B. napus* varieties, respectively (Fig. 1). Compared with the parents, the values of the F_1_ hybrids (ranging from 70 to 80) were close to the average value of both parents, indicating that the hybrids had intermediate DNA content between the parents (Fig. 1).

### Morphology characteristics of parent varieties and F_1_ hybrids

**Fig. 2:**
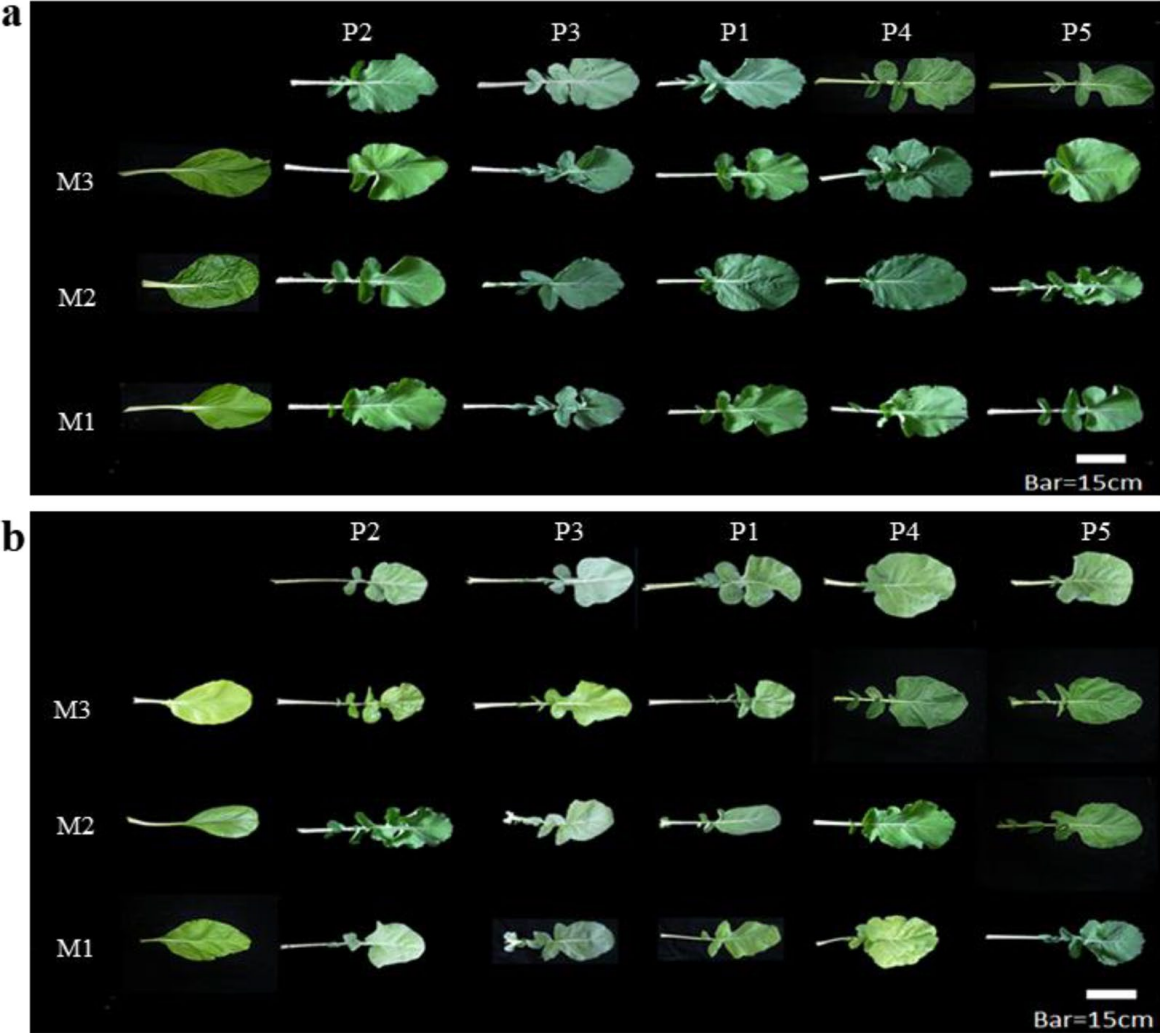
Morphology examination of rosette leaves for different varieties and the interspecific F_1_ hybrids. **a** In the experiment field. **b** In the greenhouse. M1: “Nongxing 80 days,” M2: “Wansheng rape,” M3: “Edible rape,” P1: “Deza oil No. 18,” P2: “Gueiza No. 4,” P3: “Zhong oil No. 36,” P4: “Wan oil No. 25,” and P5: “FTHEB 1001”

As shown in Fig. 2a, the rosette leaves of *B. rapa* varieties had a grassy-green color and a thin oval shape. The rosette leaves of *B. napus* varieties had grayish color, thicker lobed shape, waxy surface, and bold white veins (Fig. 2a). The lobed rosette leaves of *B. napus* varieties were jagged and notched deep to the midrib. For all F_1_ hybrids, the morphology of rosette leaves was jagged and notched, which was similar to the morphology of *B. napus* varieties in the field (Fig. 2a, Table 2). This observation was in accordance with the results of the additional examination of rosette leaves in the greenhouse (Fig. 2b). The traits of *B. napus*-like waxy surface and leaves color were also found in the rosette and stem leaves of the F_1_ hybrids (Table 2). Moreover, no lobed stem leaves in the *B. napus*, *B. rapa,* and F_1_ hybrids were observed. Most of the F_1_ hybrids had intermediate plant height and a blooming period intermediate between that of *B. napus* and *B. rapa* varieties (Table 2).

**Table 2:** Results of morphology examination of the *B. napus* and *B. rapa* varieties and the F_1_ hybrids

	Rossette leaves			Stem leaves			Plant height (cm)^c^		Blooming period (days)^d^	
	Colour^a^	Wax^b^	Lobe^b^	Colour^a^	Wax^b^	Lobe^b^				
Parents										
M1 (♀)	1 (50%)^e^	1 (66.7%)	1 (100%)	1 (96.7%)	1 (100%)	1 (100%)	129.7 ± 16.7	K^f^	13.7 ± 6.4	H
	2 (50%)	2 (33.3%)		2 (3.3%)						
M2 (♀)	1 (50%)	1 (66.7%)	1 (100%)	1 (96.7%)	1 (100%)	1 (100%)	159.7 ± 15.5	K	23.1 ± 7.1	H
	2 (50%)	2 (33.3%)		2 (3.3%)						
M3 (♀)	1 (6.7%)	1 (60%)	1 (100%)	2 (20%)	1 (100%)	1 (100%)	90.83 ± 12.6	L	19.1 ± 5.2	G
	2 (23.3%)	2 (40%)		3 (80%)						
	3 (70%)									
P1 (♂)	3 (100%)	2 (100%)	2 (100%)	3 (100%)	2 (100%)	1 (100%)	215.4 ± 12.0	FG	34.2 ± 8.8	B
P2 (♂)	3 (100%)	2 (100%)	2 (100%)	3 (100%)	2 (100%)	1 (100%)	198.9 ± 31.5	H	29.6 ± 7.9	CDE
P3 (♂)	3 (100%)	2 (100%)	2 (100%)	3 (100%)	2 (100%)	1 (100%)	201.5 ± 12.1	H	31.9 ± 7.9	BCD
P4 (♂)	3 (100%)	2 (100%)	2 (100%)	3 (100%)	2 (100%)	1 (100%)	220.7 ± 14.7	CDEFG	31.3 ± 8.4	BCD
P5 (♂)	3 (100%)	2 (100%)	2 (100%)	3 (100%)	2 (100%)	1 (100%)	166.8 ± 11.2	I	26.5 ± 5.6	EF
F_1_ hybrids (♀ × ♂)										
M1 × P1	3 (100%)	2 (100%)	2 (100%)	3 (100%)	2 (100%)	1 (100%)	228.6 ± 23.5	ABC	35.0 ± 11.9	B
M1 × P2	2 (20%)	2 (100%)	2 (100%)	3 (100%)	2 (100%)	1 (100%)	230.5 ± 16.3	AB	32.9 ± 9.1	BC
	3 (80%)									
M1 × P3	2 (10%)	2 (100%)	2 (100%)	3 (100%)	2 (100%)	1 (100%)	221.4 ± 22.8	BCDEFG	31.5 ± 6.5	BCD
	3 (90%)									
M1 × P4	3 (100%)	2 (100%)	2 (100%)	3 (100%)	2 (100%)	1 (100%)	219.4 ± 15.7	DEFG	33.9 ± 7.8	B
M1 × P5	2 (56.7%)	2 (100%)	2 (100%)	2 (20%)	2 (100%)	1 (100%)	161.9 ± 12.6	I	18.6 ± 5.6	G
	3 (43.3%)			3 (80%)						
M2 × P1	3 (100%)	2 (100%)	2 (100%)	3 (100%)	2 (100%)	1 (100%)	228.0 ± 12.1	ABCD	39.4 ± 7.9	A
M2 × P2	3 (100%)	2 (100%)	2 (100%)	3 (100%)	2 (100%)	1 (100%)	215.2 ± 18.2	FG	33.4 ± 6.7	B
M2 × P3	2 (3.3%)	2 (100%)	2 (100%)	3 (100%)	2 (100%)	1 (100%)	234.0 ± 14.6	A	34.4 ± 7.7	B
	3 (96.7%)									
M2 × P4	2 (3.3%)	2 (100%)	2 (100%)	3 (100%)	2 (100%)	1 (100%)	214.2 ± 23.9	G	33.8 ± 6.5	B
	3 (96.7%)									
M2 × P5	2 (13.3%)	2 (100%)	2 (100%)	3 (100%)	2 (100%)	1 (100%)	158.7 ± 24.7	IJ	20.9 ± 6.4	G
	3 (86.7%)									
M3 × P1	3 (100%)	2 (100%)	2 (100%)	3 (100%)	2 (100%)	1 (100%)	223.6 ± 11.8	BCDEF	28.7 ± 6.9	DEF
M3 × P2	2 (3.3%)	2 (100%)	2 (100%)	3 (100%)	2 (100%)	1 (100%)	225.2 ± 18.7	ABCDE	33.2 ± 7.4	BC
	3 (96.7%)									
M3 × P3	2 (3.3%)	1 (56.7%)	2 (100%)	2 (6.7%)	2 (100%)	1 (100%)	216.6 ± 22.3	EFG	27.0 ± 8.4	EF
	3 (96.7%)	2 (43.3%)		3 (93.3%)						
M3 × P4	3 (100%)	1 (3.3%)	2 (100%)	3 (100%)	2 (100%)	1 (100%)	198.8 ± 9.9	H	25.6 ± 8.6	F
		2 (96.7%)								
M3 × P5	1 (3.3%)	2 (100%)	2 (100%)	3 (100%)	2 (100%)	1 (100%)	151.6 ± 22.3	J	17.2 ± 5.1	GH
	2 (20%)									
	3 (76.7%)									

Morphology of “Nongxing 80 days” (M1), “Wansheng rape” (M2), “Edible rape” (M3), “Deza oil No. 18” (P1), “Gueiza No. 4” (P2), “Zhong oil No. 36” (P3), “Wan oil No. 25” (P4), “FTHEB 1001” (P5) varieties, and the resultant F_1_ hybrids were investigated in the field

^a^Rosette and stem leaf colour were grouped by RHS Colour Chart: 1 (shine green/143A), 2 (dull green/138B), and 3 (dark green/137A or N137A)

^b^Lobed leaf and wax texture were indicated as 1 (absent) or 2 (present)

^c^Plant height were investigated in full flower

^d^Blooming period were recoded from the first flowering to wilting of all flowers

^e^Percentage in the parentheses indicated the number of observed morphology divided by the total observation number (n = 30)

^f^Mean ± standard error (n = 30). Means within each column followed by the same letter(s) are not significantly different at 5% level by Fisher’s protected least significant difference test

### Identification of SRAP marker

**Fig. 3:**
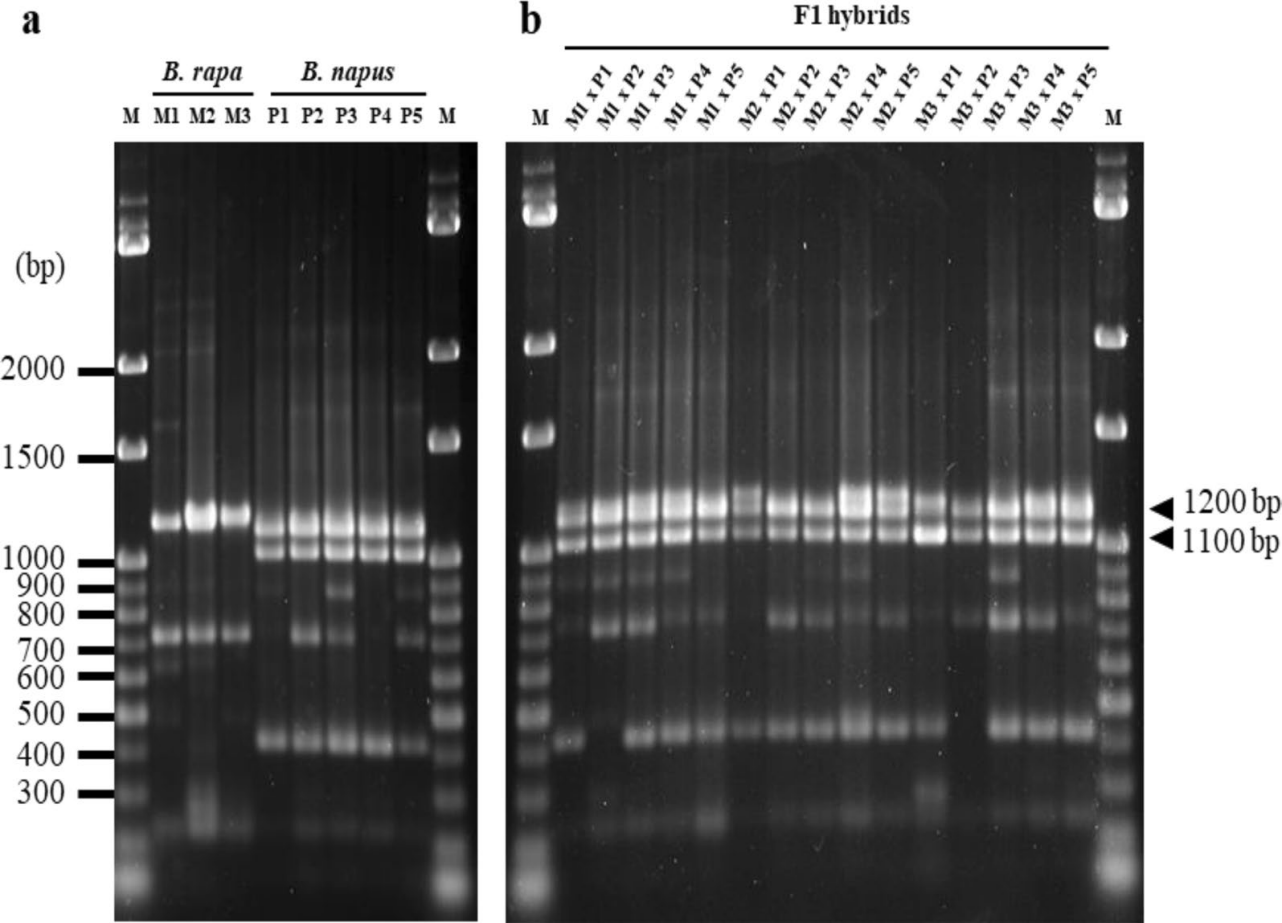
SRAP marker analysis using the me5-em1 primer set. Banding patterns for *B. rapa* and *B. napus* parents (**a**) and the F_1_ hybrids (**b**) were separated by 1.8% agarose gel. M: 100 bp marker. M1: “Nongxing 80 days,” M2: “Wansheng rape,” M3: “Edible rape,” P1: “Deza oil No. 18,” P2: “Gueiza No. 4,” P3: “Zhong oil No. 36,” P4: “Wan oil No. 25,” and P5: “FTHEB 1001.” White arrows indicate specific fragments (1.1 kb and 1.2 kb) in *B. napus* and crossed F_1_ hybrids

The SRAP marker analysis using four forward primers and four reverse primers produced bands of different sizes that ranged from 150 to 3000 bp. Among the F_1_ hybrid population, 157 bands, with an average of 152.27 bands, were produced, of which 67.87 bands were polymorphic with 44.56% polymorphism (Table 3). The number of amplified bands per primer ranged from 3 to 14, with a mean value of 9.52. We examined these DNA fragments and found that distinct bands ranging from 1.1 kb to 1.3 kb using the me5-em1 primer set could be separated on the DNA agarose gel (Fig. 3). The amplification results revealed a specific DNA pattern with an estimated molecular size of 1.3 kb in all *B. rapa* varieties (“Nongxing 80 days,” “Wansheng rape” and “Edible rape”) and two specific bands with an estimated molecular size of 1.1 and 1.2 kb in all *B. napus* varieties (“Deza oil No. 18,” “Gueiza No. 4,” “Zhong oil No. 36,” “Wan oil No. 25,” and “FTHEB 1001”) (Fig. 3a). In F_1_ hybrids, a distinct band with a molecular size of 1.1 kb and indistinguishable bands between molecular sizes of 1.2 kb and 1.3 kb were observed (Fig. 3b). The molecular data revealed that the primer combination me5-em1 could effectively identify and distinguish hybrids from their parents.

**Table 3:** SRAP marker analysis of the F_1_ hybrids

Female (♀)	Male (♂)	Total no. of bands	Average no. of bands per primer combination	Total no. of polymorphic bands	Polymorphism (%)
M1	P1	153	9.56	71	46.41
	P2	152	9.50	69	45.39
	P3	152	9.50	67	44.08
	P4	150	9.38	63	42.00
	P5	153	9.56	67	43.79
M2	P1	152	9.50	64	42.11
	P2	151	9.44	67	44.37
	P3	152	9.50	70	46.05
	P4	148	9.25	62	41.89
	P5	153	9.56	69	45.10
M3	P1	153	9.56	69	45.10
	P2	153	9.56	69	45.10
	P3	155	9.69	70	45.16
	P4	152	9.50	69	45.39
	P5	155	9.69	72	46.45
Average		152.27	9.52	67.87	44.56

Primer combination, total number of bands, average of bands per primer combination, total number of polymorphic bands, and percentage of polymorphism were calculated and listed. “Nongxing 80 days” (M1), “Wansheng rape” (M2), Edible rape (M3), “Deza oil No. 18” (P1), “Gueiza No. 4” (P2), “Zhong oil No. 36” (P3), “Wan oil No. 25” (P4), and “FTHEB 1001” (P5)

### GS and cluster analysis

**Fig. 4:**
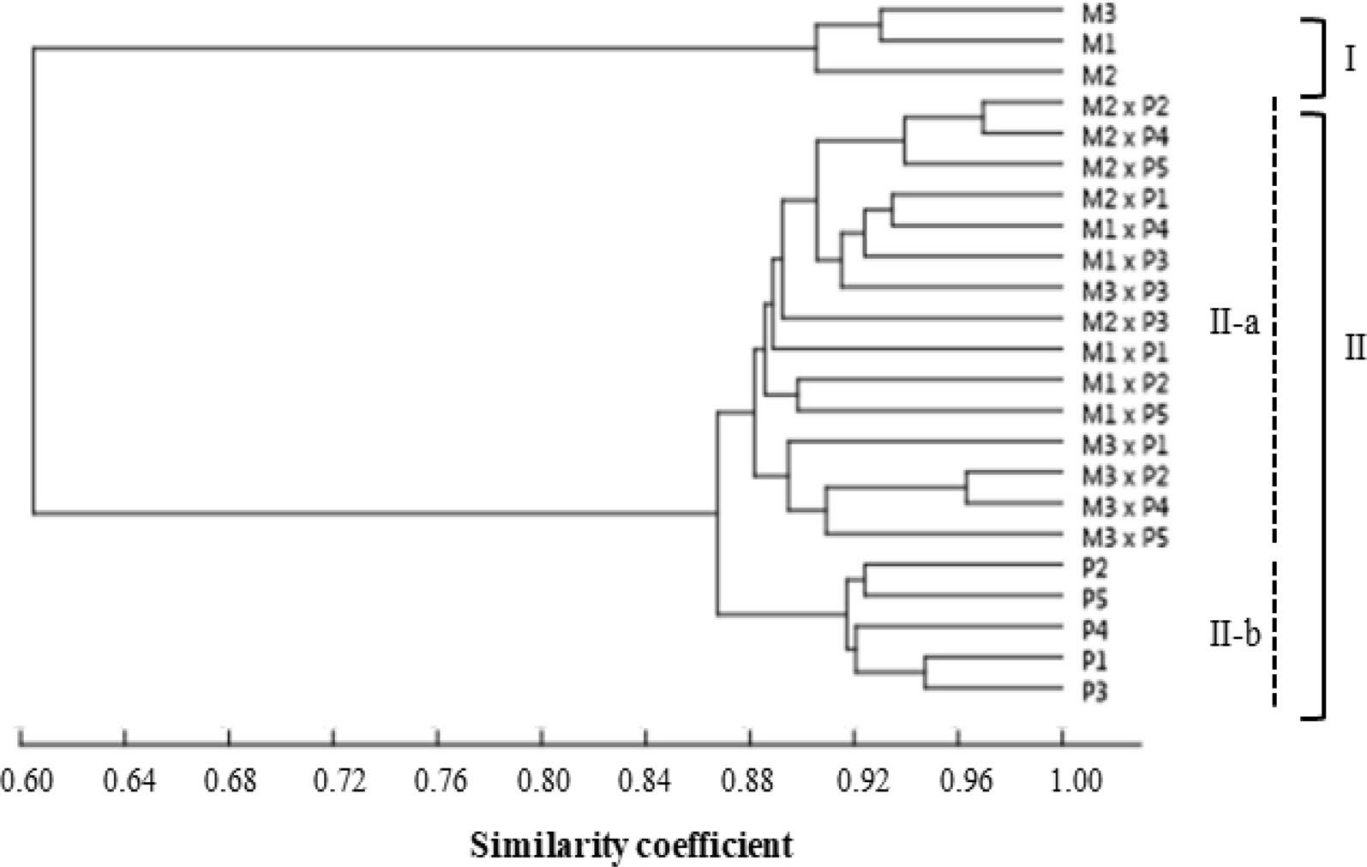
Cluster analysis based on genetic similarity between *B. rapa* and *B. napus* varieties and F_1_ hybrids. UPGMA cluster analysis was constructed using the PAST program. M1: “Nongxing 80 days,” M2: “Wansheng rape,” M3: “Edible rape,” P1: “Deza oil No. 18,” P2: “Gueiza No. 4,” P3: “Zhong oil No. 36,” P4: “Wan oil No. 25,” and P5: “FTHEB 1001”

The similarity index was calculated between different hybrids and parents based on the banding pattern. The similarity index between parents and hybrids ranged from 0.73 to 0.95 (Table 4). All F_1_ hybrids shared the majority of bands with the *B. napus* accessions (similarity index: 0.92–0.95), which were higher than the similarity with *B. rapa* accessions (similarity index 0.74–0.79) (Table 4). The cluster analysis categorized all the varieties into two main groups (Fig. 4). The first cluster included *B. rapa* “Nongxing 80 days,” “Wansheng rape,” and “Edible rape.” The second cluster was further divided into two sub-clusters II-a and II-b: II-a included 15 F_1_ hybrids, and II-b included *B. napus* “Deza oil No. 18,” “Gueiza No. 4,” “Zhong oil No. 36,” “Wan oil No. 25,” and “FTHEB 1001.” The dendrogram revealed less variation among the accessions of *B. napus* and F_1_ hybrids, suggesting that the polyploid *B. napus* (AACC) brings more diversity than the diploid *B. rapa* (AA). Therefore, F_1_ hybrids have more similarity with *B. napus*. These results demonstrated that the genetic diversity of F_1_ hybrids were similar to that of the *B. napus* varieties.

**Table 4:** Similarity index between parents and F_1_ hybrids

	M1(♀)	P1(♂)	M1 × P1	M1(♀)	P2(♂)	M1 × P2	M1(♀)	P3(♂)	M1 x P3	M1(♀)	P4(♂)	M1 x P4	M1(♀)	P5(♂)	M1 × P5
M1(♀)	1.00														
P1(♂)	0.74	1.00													
M1 × P1	0.74	0.93	1.00												
M1(♀)				1.00											
P2(♂)				0.73	1.00										
M1 × P2				0.75	0.94	1.00									
M1(♀)							1.00								
P3(♂)							0.73	1.00							
M1 × P3							0.77	0.95	1.00						
M1(♀)										1.00					
P4(♂)										0.75	1.00				
M1 × P4										0.79	0.94	1.00			
M1(♀)													1.00		
P5(♂)													0.74	1.00	
M1 × P5													0.76	0.95	1.00

	M2(♀)	P1(♂)	M2 × P1	M2(♀)	P2(♂)	M2 × P2	M2(♀)	P3(♂)	M2 × P3	M2(♀)	P4(♂)	M2 × P4	M2(♀)	P5(♂)	M2 × P5
M2(♀)	1.00														
P1(♂)	0.75	1.00													
M2 × P1	0.78	0.95	1.00												
M2(♀)				1.00											
P2(♂)				0.75	1.00										
M2 × P2				0.77	0.93	1.00									
M2(♀)							1.00								
P3(♂)							0.74	1.00							
M2 × P3							0.75	0.92	1.00						
M2(♀)										1.00					
P4(♂)										0.76	1.00				
M2 x P4										0.78	0.94	1.00			
M2(♀)													1.00		
P5(♂)													0.74	1.00	
M2 × P5													0.76	0.92	1.00

	M3(♀)	P1(♂)	M3 × P1	M3(♀)	P2(♂)	M3 × P2	M3(♀)	P3(♂)	M3 × P3	M3(♀)	P4(♂)	M3 × P4	M3(♀)	P5(♂)	M3 × P5
M3(♀)	1.00														
P1(♂)	0.74	1.00													
M3 × P1	0.76	0.92	1.00												
M3(♀)				1.00											
P2(♂)				0.73	1.00										
M3 × P2				0.78	0.93	1.00									
M3(♀)							1.00								
P3(♂)							0.74	1.00							
M3 × P3							0.77	0.93	1.00						
M3(♀)										1.00					
P4(♂)										0.73	1.00				
M3 × P4										0.77	0.93	1.00			
M3(♀)													1.00		
P5(♂)													0.73	1.00	
M3 × P5													0.75	0.93	1.00

M1: “Nongxing 80 days,” M2: “Wansheng rape,” M3: “Edible rape,” P1: “Deza oil No. 18,” P2: “Gueiza No. 4,” P3: “Zhong oil No. 36,” P4: “Wan oil No. 25,” and P5: “FTHEB 1001”

### Sequencing identification of a specific SRAP marker band

**Fig. 5:**
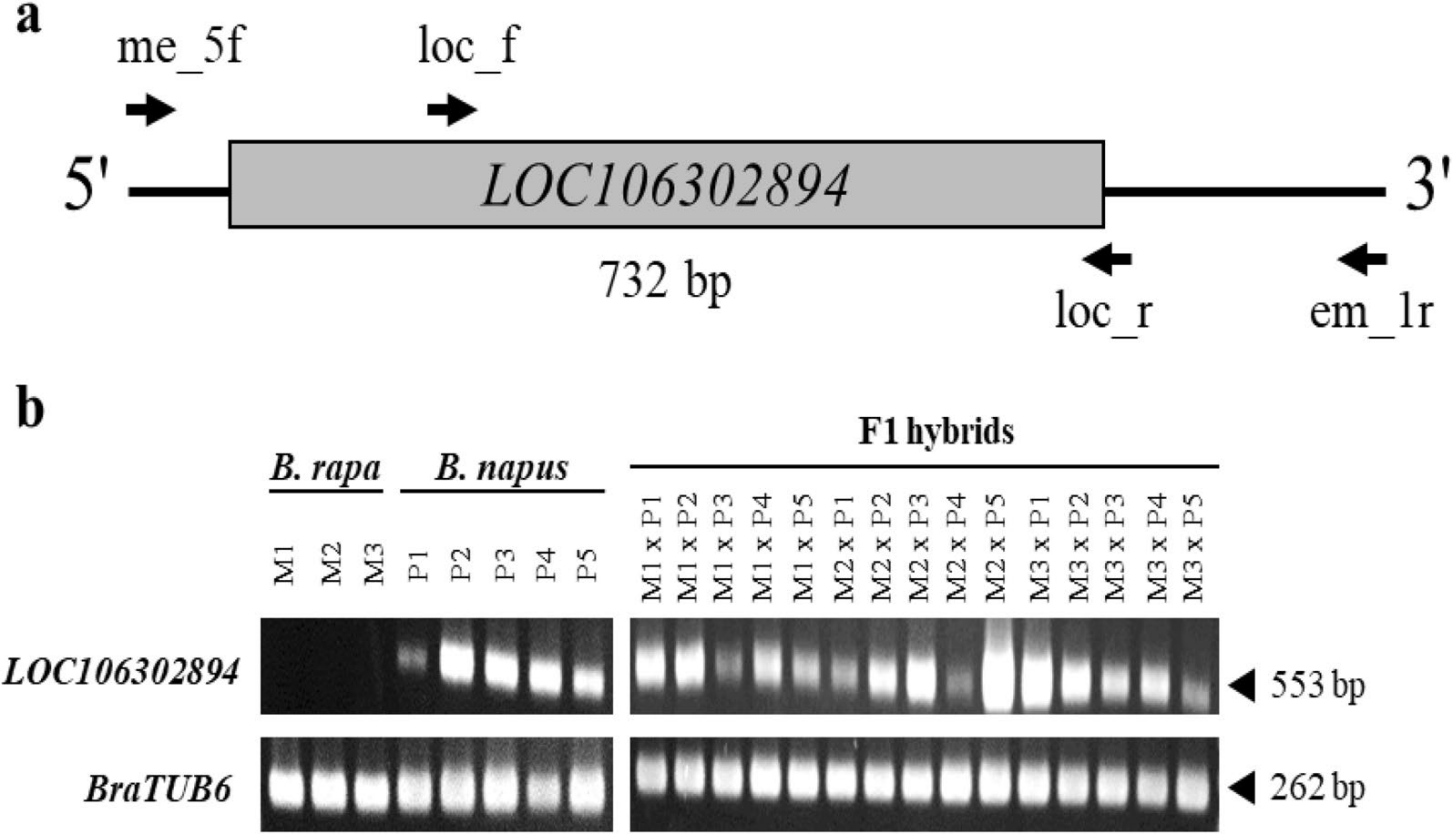
Distinguishing *B. rapa* from *B. napus* and F_1_ hybrids using the *LOC106302894* gene primer set. **a** Diagram of *LOC106302894* and position of primer set. **b** Detecting the presence of targeting in *B. rapa*, *B. napus,* and F1 hybrids. BraTUB6: internal positive control. M1: “Nongxing 80 days,” M2: “Wansheng rape,” M3: “Edible rape,” P1: “Deza oil No. 18,” P2: “Gueiza No. 4,” P3: “Zhong oil No. 36,” P4: “Wan oil No. 25,” and P5: “FTHEB 1001.”

The 1.1-kb fragment of SRAP markers was sequenced. The BLAST alignment result showed that this PCR product was similar to the reported uncharacterized *LOC106302894* gene of *B. oleracea* var. *oleracea* on chromosome C07 with 83% identities (575/689). The prediction of the translated protein (accession number XP_013594750.1) suggested that *LOC106302894* encoded a 243-amino acid protein, which includes a retrotransposon gag protein and a zinc knuckle known as the DNA-binding motif. To clarify the genetic relationship among *B. rapa*, *B. napus*, and the F_1_ hybrids, a set of primers designed based on the cloned sequences were applied. We named loc-f as the forward primer and the loc-r as a reverse primer (Table 1). The PCR amplification results revealed that a unique fragment was found from all varieties of *B. napus* and F_1_ hybrids. No amplified product was obtained from *B. rapa* varieties (Fig. 5). These results indicate that this unique primer set can successfully detect target sequences present in *B. napus* and the F_1_ hybrids (Additional file 1: Fig. S1, Additional file 2: Fig. S2, Additional file 3: Table S1).

## Discussion

This study explored the morphological and molecular characteristics that can be utilized to identify the occurrence of introgression from *B. napus* to *B. rapa* varieties in Taiwan. It has been reported that triploid F_1_ hybrids with the AAC genome (2n = 29) could be produced by interspecific hybridization from *B. napus* to *B. rapa* (Luijten et al. 2015; Sohn et al. 2016). However, the morphological characteristics and genetic relationships of *B. napus*, *B. rapa*, and their hybrid progenies remain unclear in Taiwan. Here, we first demonstrated, using FCM analysis, that the F_1_ hybrids had intermediate DNA content between the two parents (Fig. 1). Moreover, the morphology of F_1_ hybrids was similar to that of the *B. napus* varieties; furthermore, the color and the lobed shaped of the rosette leaves were similar (Table 2). The clustering and GS analysis also indicated that F_1_ hybrids were similar to *B. napus* varieties (Fig. 4).

Normally, hybrids have intermediate traits or tend to be more similar to one of their parents (Choudhary et al. 2002; Warwick et al. 2003; Niemann et al. 2015). Warwick et al. (2003) reported that the F_1_ hybrids were similar to *B. rapa* in terms of leaves, which had common color with hairs in Canada. However, in our study, the rosette leaves of F_1_ hybrids were lobed from the seedling to the flowering stage (Fig. 2). In addition, the colors of the rosette and stem leaves were more similar to those of the *B. napus* varieties (Table 2). Moreover, no variety of *B. rapa* with jagged and notched rosette leaves had been reported in Taiwan. Therefore, it was hypothesized that *B. napus*-like rosette leaves observed in the F_1_ hybrids are inherited from *B. napus* varieties. These observations were supported in early studies, which have suggested that lobed-leaf shape was a dominant trait found in *B. napus* and can be inherited by its hybrid offspring (Klein Geltink 1983; Khalid et al. 2010). Our results indicate the stable and reproducible lobed-leaf trait can be used as an early morphological characteristic to identify the F_1_ hybrids introgressed from *B. napus* and *B. rapa* in Taiwan.

Molecular markers are commonly used in crop breeding, genetic diversity analysis, and marker-assisted selection (Li et al. 2013). The occurrence of spontaneous gene flow can occur from *B. napus* to *B. oleracea*, which can be determined using the FCM and A genome-specific microsatellite marker analyses for the resultant F_1_ hybrids (Ford et al. 2006). Moreover, A and C genome-specific simple sequence repeat markers have been applied to spatially estimate the national scale of the incidence of hybridization between *B. napus* and *B. rapa* in the United Kingdon (Ford et al. 2015). In the present study, we used SRAP marker analysis to dissect the degree of separation and uniformity among the pollen donor/recipient and F_1_ hybrid varieties. We found that each primer set in F_1_ hybrids produced an average of 9.52 bands and 44.56% polymorphism (Table 3). The moderate level of polymorphic bands agreed with previous reports on *Brassica* species (Li and Quiros 2001; Ahmad et al. 2014). According to the UPGMA results (Fig. 4), the category of F_1_ hybrids was genetically identical to that of the *B. napus* varieties. This result was consistent with a higher similarity index between F_1_ hybrids and *B. napus* varieties in each hybridization combination (Table 4). Our previous study also indicated closer similarity between F_1_ hybrids and *B. napus* varieties (Hong et al. 2016).

Compared with the other types of molecular markers, our results indicated that the SRAP marker analysis provides a more efficient and robust way, with less labor, to identify hybridization between two similar species and its relatives. To establish a fast identification method using the SRAP marker analysis, we selected the unique 1100-bp amplicon found only in *B. napus* varieties and the F_1_ hybrids. The most repeatable and reproducible one with 1100-bp band was homologous to the *LOC106302894* putative gene and located on the chromosome C07 of *B. oleracea var. oleracea*. This finding correlated with the triangle of U, which suggested that the genome in *B. napus* originated through spontaneous interspecific hybridization between its ancestral diploid species *B. rapa* (AA) and *B. oleracea* (CC) (Nagaharu 1935). Reports have indicated that triploid AAC F_1_ hybrids produced from *B. rapa* (♀) × *B. napus* (♂) caused the univalent C chromosomes (Choudhary et al. 2002; Leflon et al. 2006). We suggested that the 1100-bp band was located in the C genome and specific to identify the F_1_ hybrids introgressed from *B. napus* to *B. rapa* at the seedling stage.

## Conclusion

The specific *B. napus* amplicon *LOC106302894* and the distinct morphological trait lobed rosette leaves were found in all F_1_ hybrids but not in the pollen recipient—*B. rapa* varieties. These genetic and phenotypic characteristics can be used for efficiently identify the interspecific hybrids from *B. rapa *×* B. napus* varieties in an early stage to prevent and monitor the occurrence of unintentional introgression in Taiwan. Furthermore, Ford et al. (2006) used A genome-specific marker to conduct a preliminary assessment of the ecological impact of the gene flow from *B. napus* to *B. oleracea*. Similarly, we believe the C genome-specific marker *LOC106302894* can be used to assess the extent of gene flow from *B. napus* to *B. rapa* in Taiwan.

## Supplementary information

**Additional file 1: Fig. S1.** Screening the *LOC106302894* among common *Brassicaceae* vegetables in Taiwan. (a) Theory of ‘Triangle of U’ in *Brassica* species (Nagaharu 1935). (b) and (c) PCR amplification results using LOC106302894 and BraTUB6 primer. Banding patterns were separated in 1.8% agarose gel. Genomic DNA of classic CC genome species *Brassica oleracea* such as broccoli (*B. oleracea* var. italica), cabbage (*B. oleracea* var. capitata), cauliflower (*B. oleracea* var. botrytis), and Chinese kale (*B. oleracea* var. alboglabra) were analyzed. Other *Brassicaceae* relatives including one AABB genome species leaf mustard (*B. juncea* (L.) Czern.), two AA genome species bok-choy (*B. rapa* var. Chinensis) and Chinese cabbage (*B. rapa* var. pekinensis), and white radish (*Raphanus sativus* var. Longipinnatus) were also investigated.

**Additional file 2: Fig. S2.** BLAST and alignment results of the 1100 bp fragment. The identity and consensus positions of sequence *B. oleracea* HDEM genome scaffold C5 (LR031877.1), *B. oleracea* HDEM genome scaffold C7 (LR031876.1) and LOC101602894 (XP_013594750.1) are labeled in yellow and blue text background respectively. Primer location including SRAP (me5f and em1r) and LOC101602894 (Loc_f and Loc_r) was also highlighted in red-color arrows.

**Additional file 3: Table S1.** Nucleotide BLAST results of 1.1 kb amplicons. The sequence of the 1.1 kb fragment has 95.6%, 82.8% and 90.0% identity with C5, C7 and C2 scaffold DNA of the *B. oleracea*. In addition, it was found the 1.1 kb fragment has 83.5% identity with LOC106302894 mRNA of *B. oleracea*.

## Data Availability

The data and materials used and analyzed in the current study can be provided from the corresponding author for scientific, non-profit purpose.

## Abbreviations

GM: genetically modified
SRAP: sequence-related amplified polymorphism
FCM: flow cytometry
AFLP: amplified fragment length polymorphism
ORFs: open reading frames
UPOV: International Union for the Protection of New Varieties of Plants
DUS: distinctness, uniformity and stability
PA: Ploidy Analyser
GS: genetic similarity
UPGMA: unweighted pair group method with arithmetic mean
BLAST: basic local alignment search tool
*BraTUB6*: *Brassica tubulin beta*-*6*
