# Supplementary material for: Morphological and genetic characteristics of F1 hybrids introgressed from Brassica napus to B. rapa in Taiwan
Source: Bot Stud. 2020 Jan 21;61:1. doi: 10.1186/s40529-019-0279-5 (PMC6974233; doi:10.1186/s40529-019-0279-5)
Supplement: Supplementary file 1 — Additional file 1: Fig. S1. Screening the LOC106302894 among common Brassicaceae vegetables in Taiwan. (a) Theory of ‘Triangle of U’ in Brassica species (Nagaharu 1935). (b) and (c) PCR amplification results using LOC106302894 and BraTUB6 primer. Banding patterns were separated in 1.8% agarose gel. Genomic DNA of classic CC genome species Brassica oleracea such as broccoli (B. oleracea var. italica), cabbage (B. oleracea var. capitata), cauliflower (B. oleracea var. botrytis), and Chinese kale (B. oleracea var. alboglabra) were analyzed. Other Brassicaceae relatives including one AABB genome species leaf mustard (B. juncea (L.) Czern.), two AA genome species bok-choy (B. rapa var. Chinensis) and Chinese cabbage (B. rapa var. pekinensis), and white radish (Raphanus sativus var. Longipinnatus) were also investigated. [file 40529_2019_279_MOESM1_ESM.docx]

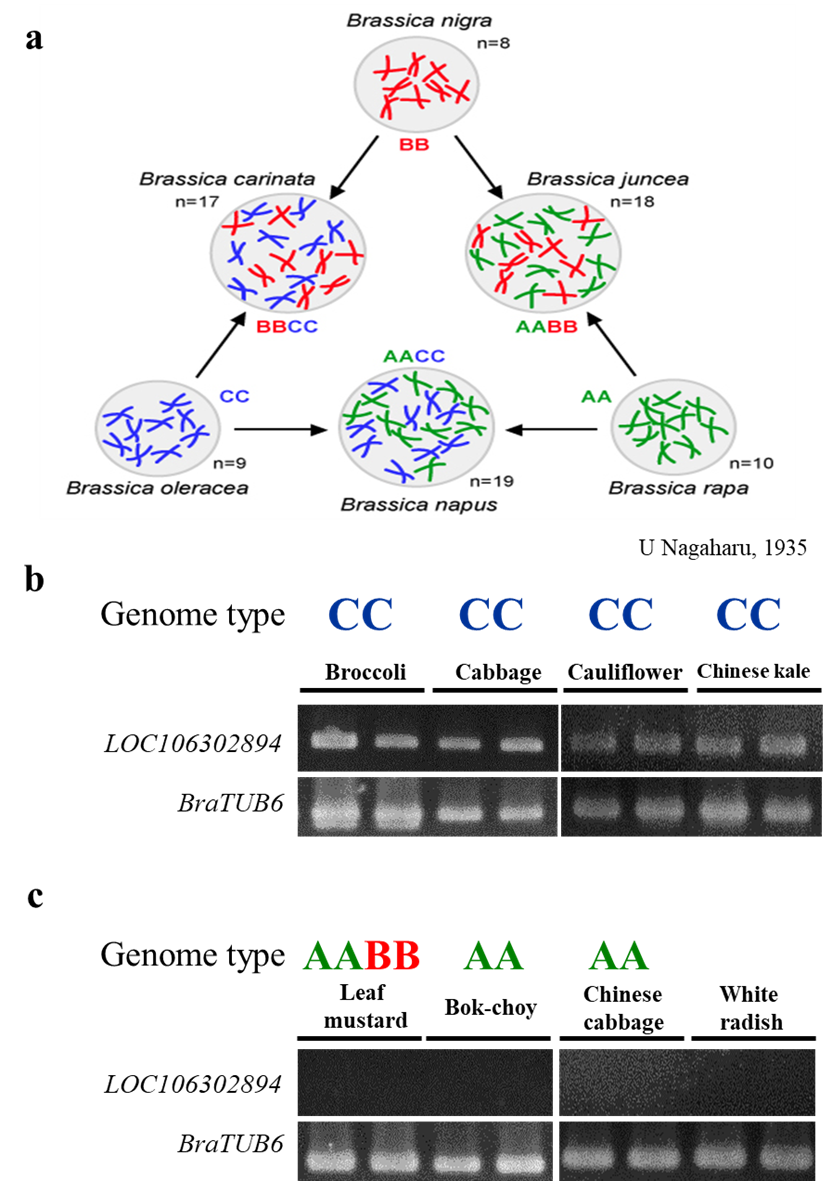


**Fig. S1.** Screening the *LOC106302894* among common *Brassicaceae* vegetables in Taiwan. (a) Theory of ‘Triangle of U’ in *Brassica* species (U 1935). (b) and (c) PCR amplification results using LOC106302894 and BraTUB6 primer. Banding patterns were separated in 1.8% agarose gel. Genomic DNA of classic CC genome species *Brassica oleracea* such as broccoli (*B. oleracea* var. italica), cabbage (*B. oleracea* var. capitata), cauliflower (*B. oleracea* var. botrytis), and Chinese kale (*B. oleracea* var. alboglabra) were analyzed. Other *Brassicaceae* relatives including one AABB genome species leaf mustard (*B. juncea* (L.) Czern.), two AA genome species bok-choy (*B. rapa* var. Chinensis) and Chinese cabbage (*B. rapa* var. pekinensis), and white radish (*Raphanus sativus* var. Longipinnatus) were also investigated.
