# Supplementary material for: Morphological and genetic characteristics of F1 hybrids introgressed from Brassica napus to B. rapa in Taiwan
Source: Bot Stud. 2020 Jan 21;61:1. doi: 10.1186/s40529-019-0279-5 (PMC6974233; doi:10.1186/s40529-019-0279-5)
Supplement: Supplementary file 2 — Additional file 2: Fig. S2. BLAST and alignment results of the 1100 bp fragment. The identity and consensus positions of sequence B. oleracea HDEM genome scaffold C5 (LR031877.1), B. oleracea HDEM genome scaffold C7 (LR031876.1) and LOC101602894 (XP_013594750.1) are labeled in yellow and blue text background respectively. Primer location including SRAP (me5f and em1r) and LOC101602894 (Loc_f and Loc_r) was also highlighted in red-color arrows. [file 40529_2019_279_MOESM2_ESM.docx]

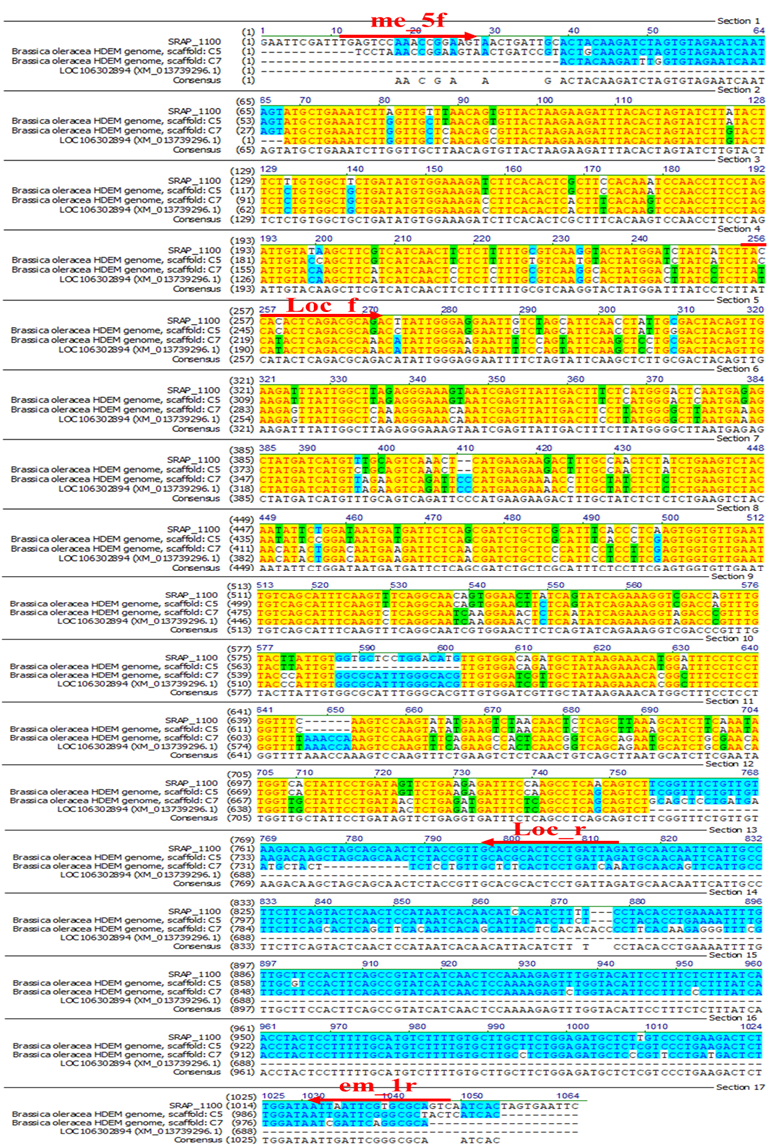


**Fig. S2** BLAST and alignment results of the 1100 bp fragment. The identity and consensus positions of sequence *B. oleracea* HDEM genome scaffold C5 (LR031877.1), *B. oleracea* HDEM genome scaffold C7 (LR031876.1) and LOC101602894 (XP_013594750.1) are labeled in yellow and blue text background respectively. Primer location including SRAP (me5f and em1r) and LOC101602894 (Loc_f and Loc_r) was also highlighted in red-color arrows.
