# Supplementary material for: Morphological and genetic characteristics of F1 hybrids introgressed from Brassica napus to B. rapa in Taiwan
Source: Bot Stud. 2020 Jan 21;61:1. doi: 10.1186/s40529-019-0279-5 (PMC6974233; doi:10.1186/s40529-019-0279-5)
Supplement: Supplementary file 3 — Additional file 3: Table S1. Nucleotide BLAST results of 1.1 kb amplicons. The sequence of the 1.1 kb fragment has 95.6%, 82.8% and 90.0% identity with C5, C7 and C2 scaffold DNA of the B. oleracea. In addition, it was found the 1.1 kb fragment has 83.5% identity with LOC106302894 mRNA of B. oleracea. [file 40529_2019_279_MOESM3_ESM.docx]

| Description | Accession | Max score | Total score | Query cover | E value | Per. Ident |
| --- | --- | --- | --- | --- | --- | --- |
| *Brassica oleracea* HDEM genome, scaffold: C5 | LR031877.1 | 1639 | 1639 | 97% | 0.0 | 95.64% |
| *Brassica oleracea* HDEM genome, scaffold: C7 | LR031876.1 | 876 | 876 | 94% | 0.0 | 82.79% |
| PREDICTED: *Brassica oleracea* var. oleracea uncharacterized LOC106302894 (LOC106302894), mRNA | XM_013739296.1 | 630 | 630 | 64% | 3e-176 | 83.45% |
| *Brassica oleracea* HDEM genome, scaffold: C2 | LR031874.1 | 401 | 401 | 29% | 3e-107 | 90.00% |
| *Arabis alpina* genome assembly, chromosome: 1 | LT669788.1 | 62.1 | 62.1 | 3% | 5e-05 | 100.00% |

**Table S1.** Nucleotide BLAST results of 1.1 kb amplicons. The sequence of the 1.1 kb fragment has 95.6%, 82.8% and 90.0% identity with C5, C7 and C2 scaffold DNA of the *B. oleracea*. In addition, it was found the 1.1 kb fragment has 83.5% identity with LOC106302894 mRNA of *B. oleracea.*
